# Cardiac Troponin Biosensor Designs: Current Developments and Remaining Challenges

**DOI:** 10.3390/ijms23147728

**Published:** 2022-07-13

**Authors:** Andreea Campu, Ilinca Muresan, Ana-Maria Craciun, Simona Cainap, Simion Astilean, Monica Focsan

**Affiliations:** 1 Nanobiophotonics and Laser Microspectroscopy Center, Interdisciplinary Research Institute in Bio-Nano-Sciences, Babes-Bolyai University, Treboniu Laurian No. 42, 400271 Cluj-Napoca, Romania; andreea.campu@ubbcluj.ro (A.C.); ilinca.muresan@stud.ubbcluj.ro (I.M.); ana.gabudean@ubbcluj.ro (A.-M.C.); simion.astilean@ubbcluj.ro (S.A.); 2 Department of Pediatric Cardiology, Pediatric Clinic 2, Emergency County Hospital for Children, Crisan No. 3-5, 400124 Cluj-Napoca, Romania; cainap.simona@gmail.com; 3 Department of Mother & Child, Iuliu Hatieganu University of Medicine and Pharmacology, Louis Pasteur No. 4, 400349 Cluj-Napoca, Romania; 4 Biomolecular Physics Department, Faculty of Physics, Babes-Bolyai University, Mihail Kogalniceanu No. 1, 400084 Cluj-Napoca, Romania

**Keywords:** cardiac troponin biomarkers, surface plasmon resonance, surface enhanced Raman spectroscopy, fluorescence detection, electrochemical detection

## Abstract

Acute myocardial infarction (AMI) is considered as one of the main causes of death, threating human lives for decades. Currently, its diagnosis relies on electrocardiography (ECG), which has been proven to be insufficient. In this context, the efficient detection of cardiac biomarkers was proposed to overcome the limitations of ECG. In particular, the measurement of troponins, specifically cardiac troponin I (cTnI) and cardiac troponin T (cTnT), has proven to be superior in terms of sensitivity and specificity in the diagnosis of myocardial damage. As one of the most life-threatening conditions, specific and sensitive investigation methods that are fast, universally available, and cost-efficient to allow for early initiation of evidence-based, living-saving treatment are desired. In this review, we aim to present and discuss the major breakthroughs made in the development of cTnI and cTnT specific biosensor designs and analytical tools, highlighting the achieved progress as well as the remaining challenges to reach the technological goal of simple, specific, cheap, and portable testing chips for the rapid and efficient on-site detection of cardiac cTnI/cTnT biomarkers in order to diagnose and treat cardiovascular diseases at an incipient stage.

## 1. Introduction

Acute myocardial infarction (AMI) is the most severe cardiovascular disease, which has threatened human lives for decades, becoming, consequently, one of the main causes of death according to the World Health Organization (WHO) [1]. Unfortunately, the WHO report estimates that over 23.3 million people will die annually from cardiovascular diseases by 2030 [2]. Nowadays, although electrocardiography (ECG) is the commonly employed tool for the diagnosis of AMI, only 57% of patients can be correctly diagnosed using this technique [3]. In this context, the early diagnosis of AMI is crucial for the early initiation of evidence-based treatment. There is a continuous interest in finding cardiac biomarkers to predict risk and, implicitly, fulfil the early diagnosis requirements in the AMI setting. Biomarkers, as measurable indicators of a biological state, can successfully detect pathogenic processes, and therefore, cardiac troponins, myoglobin, and creatine kinase-MB represent, at this moment, valuable diagnostic biomarkers for AMI [4].

Furthermore, the efficient and accurate detection of cardiac biomarkers in the incipient stage of AMI can greatly impact the progress of precision medicine, allowing the prescription of personalized treatment with regard to the necessities of each patient. This innovative concept takes into consideration the genetics, environment, and lifestyle of the patients to tailor both their treatment and diagnosis approaches. In this context, highly specific and sensitive biosensors could provide a significant amount of information, leading to the development of adequate cardio protection strategies [5,6,7]. Moreover, in the context of personalized diagnostics and therapy, a key factor in their determination is represented by the sex of the patient. AMI was shown to have a higher incidence rate for women than men [8]. To fill the male–female sex gap, when characterizing cardiovascular diseases, specific biomarker differences should be highlighted with precision, thus making the development of highly accurate sex-specific cardiac biomarker biosensors even more desirable.

Conventionally, AMI used to be diagnosed based on typical clinical settings, ST segment, and T wave abnormalities on the ECG and an increase in cardiac muscle enzymes (CK-MB). However, when it was shown that not all patients with AMI presented significantly increased cardiac enzymes, a different biological marker was sought. Cardiac troponins (cTn), in particular, are a group of proteins found in the skeletal and cardiac muscle fibers, which enable muscular contraction. The muscle contraction is a very complex event, requiring the specific interaction between the actin and myosin filaments present in the muscle fibers. This interaction is mediated by the troponin–tropomyosin complex. There are three different types of troponin proteins, each with a specific role: troponin C, T, and I. Troponin C (cTnC) binds calcium ions in the sarcoplasm, thus initiating the muscular contraction. Troponin I (cTnI) is the inhibitory subunit of the troponin complex which, by binding to the actin filament, prevents the muscle contraction. Troponin T (cTnT) binds to tropomyosin (a helicald-coiled protein wound around the actin filaments), therefore anchoring the troponin complex to the muscle fiber, leading to the shortening of the muscle fiber and contraction [9]. This is how the measurement of serum cTnI and cTnT developed, which proved to be superior in terms of sensitivity and specificity to the cardiac muscle enzyme measurements in the diagnosis of myocardial damage. Increased cardiac troponin concentrations are now regarded as standard biochemical markers for the diagnosis of AMI [10]. As one of the most life-threatening conditions, the AMI diagnosis requires specific and sensitive investigation methods, which should be fast, universally available, and cost-efficient, in order to allow for the early initiation of evidence-based, living-saving treatment. Measuring the amount of cTnT and/or cTnI in blood is one of the cornerstones in the diagnosis of myocardial disease [11].

The main method used today for the detection and quantification of troponin is via electrochemiluminescence or an electro-generated chemiluminescence immunoassay (ECLIA), a powerful and versatile detection method for many biomarkers [12,13]. However, even if ECLIA might be useful in detecting troponin levels, it presents some disadvantages, one of the biggest being its high costs. ECLIA is an expensive method that also requires a well-equipped laboratory with sophisticated equipment and trained technicians [14]. In terms of sensitivity, every detection assay comes with its own reference range, however, the coefficient of variation (CV) for a given kit should be below 10% in order to be approved. The sensitivity, specificity, and precision of the different commercially available troponin assays vary considerably. These differences are related to a lack of standardization, the use of different monoclonal antibodies, the presence of modified cTnI and cTnT in the serum, and variations in antibody cross-reactivity to the various detectable forms of cTnI that result from its degradation [10]. Recently, new molecules have been approved for the diagnosis of AMI using cTn: highly-sensitive cTnI and hs-cTnT. These tests revealed an increased sensibility of up to 96%, compared to the old, standard tests, which had a sensibility of only to 90% [11]. In line with the above-mentioned requirements, an enormous amount of effort has been dedicated to the development of simple, specific, cheap, and portable testing chips for the rapid and efficient on-site detection of cTnI/cTnT biomarkers in order to diagnose and treat cardiovascular diseases at an incipient stage. In this review, we aim to provide an overview of the advances made in the development of cTnI and cTnT biosensors using different analytical methods in order to achieve high sensitivity and specificity. The advantages and drawbacks of representative biosensor designs are discussed, highlighting the progress made in achieving high-throughput detection systems. Further challenges are also presented, that once overcome could lead to personalized treatment and thus greatly impact the health care system.

## 2. Surface Plasmon Resonance-Based Biosensors

Recently, the development of sensitive, rapid, efficient, and specific devices for the detection of cardiac troponin biomarkers has gathered a lot of interest. In this context, surface plasmon resonance (SPR)-based platforms have been developed and successfully implemented in the diagnosis of AMI. SPR sensors exploit electromagnetic waves that cause changes in the refractive index of the environment close to the metallic nanoparticles and nanostructured films when a molecule is adsorbed onto their surface. The appeal of the method relies in its label-free detection of biomolecules, so it can be used to track the interactions between analytes in a direct manner [15]. Human cardiac troponins are inhibitory proteins that are released quickly after an acute myocardial infarction and that increase in concentration remains in the blood for 4 days after. Some of the disadvantages such as long diagnostics time, high cost, and low biosensitivity exhibited by traditional techniques such as radioimmunoassay or enzyme-linked immunosorbent assay (ELISA) can be overcome using SPR detection [1,16].

**Figure 1:**
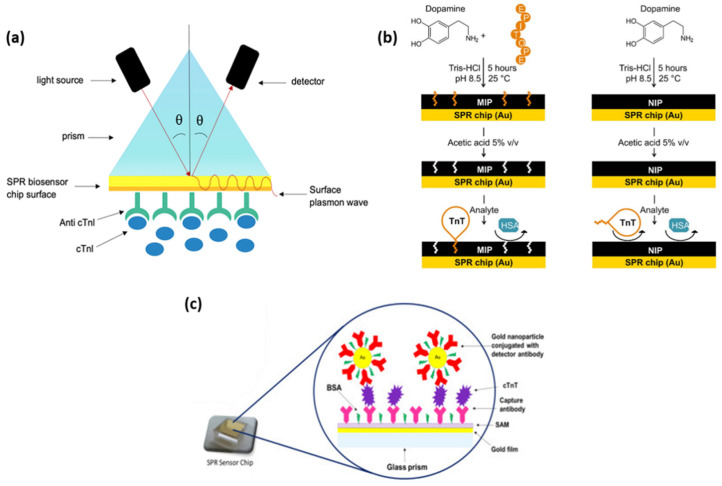
A schematic illustration of the SPR analysis principle for the cTnI biomarker detection (**a**), dopamine MIP and NIP for the specific capture and detection of cTnT (**b**), and direct and sandwich assay methods for the detection of cTnT using AuNPs (**c**). Figure adapted from references [19,20,22].

Based on the simple SPR principle, Masson et al., developed a fiber-optic-based SPR sensor for the specific and sensitive detection of the cardiac biomarkers myoglobin (MG) and cTnI, respectively [17]. The gold (Au) surface at the tip of the probe was functionalized and activated to bind the specific antibodies for MG and cTnI by amine coupling. The biomarkers were efficiently detected from a buffered aqueous solution of both target analytes by dipping the fiber-optic-based sensor. The final aim was the in vivo detection of AMI, thus the SPR sensor was optimized for biologically relevant pH and temperature, for which limits of detection of 2.9 ng/mL for MG and, respectively, 1.4 ng/mL for cTnI were achieved. Additionally, the high stability of the antibody functionalized probe surface over a few weeks at pH ranging from 2 to 12 constitutes a considerable advantage with respect to other methods. Another SPR sensor constructed on a modified Au thin film was demonstrated to efficiently detect cTnI up to 68 pg/mL by monitoring the SPR angle shift [18]. For the specific capture of the cardiac biomarker, a peptide epitope was used to generate highly specific anti-cTnI monoclonal antibodies (Mab) in mouse ascites, which were then extracted and used to functionalize the Au thin film in light of the cTnI capture. Au chip SPR biosensors exhibit high sensitivity, real-time detection, and low sample volumes as well as ultralow limits of detection (LOD), as demonstrated by Cimen et al., who efficiently implemented an immobilized anti-cTnI Mab SPR biosensor for the detection of cTnI in real-time from aqueous solutions and patient blood samples with high reproducibility and long-term stability (up to 27 months) [19]. The Au chip surface was activated and the anti-cTnI antibody was grafted (Figure 1a), thus obtaining the high selectivity demonstrated by the competitive adsorption of cTnI, MG, immunoglobulin G (IgG), and prostate specific antigen (PSA). By employing the proposed immunoassay, a LOD of 0.12 pg/mL was calculated along with a quantification limit of 0.41 pg/mL. Furthermore, the technology shows great potential to be used as a cell surfaceome array in the identification of cardio-specific biomarkers. Along with cTnI, cTnT is released in the blood stream from the cardiac tissue as a consequence of myocardial damage. Therefore, its specific detection could significantly improve the accuracy of AMI diagnostics. Nanomolar levels of cTnT were successfully reached by SPR detection using a polydopamine (PDA) film imprinted with the specific cTnT-epitope [20]. The molecularly imprinted polymer (MIP) receptor was realized by generating a spontaneous self-assembling process of dopamine in an alkaline aqueous environment and imprinting short cTnT-derived peptides as a recognition element (Figure 1b). Its rapid growth on the Au sensor surface, which takes only a few hours, allows it to avoid the previously observed immobilization of antibodies. SPR label-free molecule detection in real-time has been performed by measuring the change in the refractive index at the MIP film upon analyte binding. Film selectivity and protein/surface non-specific interactions were demonstrated using human serum albumin (HSA). Moreover, cTnT on non-imprinted polymer (NIP) dopamine was tested, proving the superiority of the proposed epitope-imprinted polymer. A key aspect in the development of diagnostic/analytical tools is the anti-fouling ability of the biosensor. To avoid non-specific binding and, implicitly, false positive or negative responses, Liu et al. designed a SPR biosensor based on a self-assembled monolayer of a homogeneous mixture of oligo (ethylene glycol) (OEG)-terminated alkanethiolate and mercaptohexadecanoic acid (MHDA) on Au to immobilize the cTnT antibodies [21]. The homogeneous densely packed and smooth surface showed no phase segregation, which enables the detection of cTnT via SPR biosensing up to a LOD of 100 ng/mL within 2 min after injection. The high resistance to HSA confirms the anti-fouling ability of the as-designed biosensor.

With the advancement of nanotechnology and, implicitly, the development of nanomaterials, SPR-based immunoassays for cardiac biomarker detection have taken great benefit from employing metallic nanoparticles of various compositions, shapes, and sizes, thus leading to the emergence of diagnostic tools incorporating multiple technologies. Using a new SPR-based sensor, both direct and sandwich assay methods for the detection of cardiac troponin T were explored and optimized by Pawula et al. [22]. The introduction of spherical gold nanoparticles (AuNPs) attached to the detecting antibody improved the reaction much further (Figure 1c). To allow the sensor to be reused numerous times, a regeneration technique was devised. For direct and sandwich experiments in buffer, the SPR immunosensor demonstrated high repeatability and reliability for cTnT measurements in the concentration range of 25–1000 ng/mL and 5–400 ng/mL. For both tests, a linear regression analysis was conducted, and the R^2^ value was determined to be 0.99. A LOD of 0.5 ng/mL in the linear dynamic range of 0.5–40 ng/mL was reached for 50% of the actual serum samples. Another type of nanostructure that was proven to be effective in the development of targeted cardiac biomarker detection is hollow Au nanoparticles (HAuNPs). Wang et al. developed a sandwich-like immunoassay based on HAuNP-enhanced SPR for the detection of cTnI [23]. The surface of a Au chip was efficiently modified with HAuNPs and a thin layer of PDA allowing for the direct immobilization of the cTnI antibodies. After the capture of the cTnI biomarker, a secondary antibody was introduced to finalize the sandwich assay. The use of HAuNPs was found to strongly enhance the SPR signal proportionally with the cTnI concentrations from 1.25 to 40 µg/mL, however, the LOD was considerably smaller, 38 ng/mL, respectively. Similarly, Song and collaborators designed an ultrasensitive magnetic field-assisted SPR immunoassay to efficiently isolate and detect cTnI [16]. On the HAuNP-modified Au thin film, a PDA layer was formed to ensure the capture of antibody immobilization. However, in this specific design, the authors conjugated PDA-wrapped magnetic multi-walled carbon nanotubes (MMWCNT-PDA) with the detection antibodies in order to ensure the magnetic extraction of the cTnI from the sample, thus operating as fast “vehicles” with a high cTnI loading and extraction efficiency. The proposed system reached a LOD of 1.25 ng/mL. Moreover, the magnetic component overcame the slow diffusion-limited mass transfer and matrix interference effect, leading to an improved sensitivity of the biosensor. Similarly, Sun et al. developed an immunomagnetic SPR assay for cTnI detection following the same principle [24]. However, in their work, the Au film was modified with AuNPs having an average diameter of 20 nm and PDA to further immobilize the captured anti-cTnI. Concomitantly, core-shell Fe_3_O_4_@PDA nanohybrids were prepared as magnetic elements and conjugated with detection antibodies to form the immune probes, which specifically collect cTnI by magnetic bioseparation. After the capture of cTnI by the sensing platform, MMWCNT-PDA functionalized with silver nanoparticles (AgNPs) and conjugated with a secondary antibody were introduced to further enhance the SPR signal, thus reaching a LOD of 3.75 ng/mL.

Furthermore, due to their unique optical properties related, in particular, to the high sensitivity of the localized surface plasmon resonance (LSPR) to refractive index changes in the environment, anisotropic nanoparticles are excellent signal transducers. Guo and collaborators developed a method to detect cTnI in solution by monitoring the LSPR of gold nanorods (AuNRs) [25]. The specific capture of cTnI by its specific human anti-cTnI changes the dielectric environment and thus induces a change in the LSPR position. High aspect ratio AuNRs have been validated as efficient signal transducers in the proposed immunoassay by enabling a LOD of 10 ng/mL, holding a much higher sensitivity than spherical nanostructures. Furthermore, AuNRs have been demonstrated to provide a visual detection of cTnI in the case of non-enzymatic colorimetric sensors. Recently, Raj et al. conducted a study that implies the aggregation of AuNRs with heparin [26]. Concretely, CTAB-stabilized AuNRs are exposed to heparin, which, due to electrostatic interaction, induces an aggregation of the AuNRs and, implicitly, a reduction in the LSPR wavelength. This process can also be observed visually, as the colloidal solution changes its color from red to blue. When exposed to cTnI, the electrostatic equilibrium of the obtained plasmonic system will be disrupted, considering that the tendency to form heparin and cTnI complexes is stronger than the electrostatic interaction between heparin and AuNRs. Thus, the aggregation of the AuNRs will be reversed, which can be translated as a change in the LSPR position to higher wavelengths as well as a color change of the solution. With increasing the concentration of cTnI, the color progressively returns to red. The proposed non-enzymatic colorimetric sensor enables the detection of the cardiac biomarker up to 0.4 ng/mL.

SPR-based biosensors are able to detect target biomarkers in a simple, label-free, and fast manner, providing straightforward information if the interaction between the capture probe and target analyte is realized based on refractive index changes. Moreover, SPR biosensors are already implemented in point-of-care (PoC) devices due to their versatility, long-term stability, and simple concepts, which enable their integration into miniaturized portable devices able to operate as efficient diagnostic tools. Despite the significant progress in their development, the sensitivity of the SPR biosensors for the detection of cardiac troponin I and T still needs to be improved to reach values sufficient for clinical diagnosis.

## 3. Surface Enhanced Raman Spectroscopy (SERS) Sensing Platforms

Recently, a significant amount of effort has been dedicated to enable the ultrasensitive detection of cardiac troponin biomarkers by developing surface-enhanced Raman spectroscopy (SERS) biosensing point-of-care (POC) devices. As an important vibrational analysis technique, the Raman spectroscopy provides a set of information regarding the chemical structure of a molecule due to its “fingerprinting” capabilities, which allows its specific identification. The Raman effect is known to be a weak phenomenon [27], hence, to overcome this major drawback, the Raman signal is enhanced by placing the target molecules close to a metallic surface giving rise to SERS. The scientific community describes the SERS mechanism by two accepted theories [28]: the electromagnetic enhancement (EM), which arises due to the resonant photoexcitation of the metallic surface plasmons, thus significantly amplifying the electromagnetic field, and chemical mechanism (CM), arising from the charge transfer between the absorbate and metallic surface inducing changes in the polarizability of the molecules. EM is responsible for 10^12^ enhancement factors [29,30,31], whereas CM reaches 10^2^–10^4^ orders of magnitude contingent upon the target absorbed species [32]. SERS is a suitable analytical tool for a wide range of applications, hence various types of SERS-active substrates have been developed. Commonly, SERS substrates have been obtained by the deposition of metallic nanoparticles on a solid substrate, however, in recent years, the designs of SERS-active substrates have become more intriguing and advantageous [33,34].

**Figure 2:**
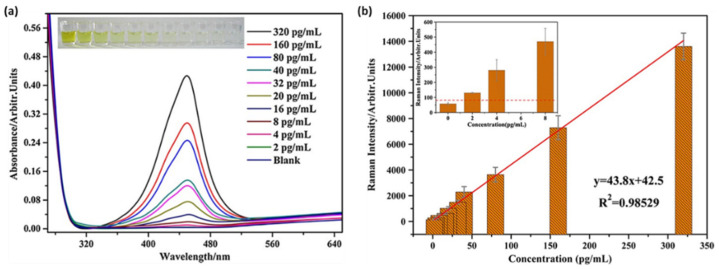
(**a**) The absorption spectra of the TMB^2+^ in the presence of different cTnT concentrations (from 2 to 320 pg/mL). A digital image of the ELISA plate is shown in the inset. (**b**) Plot of the SERS signal intensity against the cTnT concentration. The inset presents a zoom of the 0 to 8 pg/mL cTnT concentration range. Figure adapted from Yu et al. [35].

The cardiac troponin biomarker is generally detected by ELISA, which is a traditional enzyme-catalyzed detection technique based on colorimetric readings. Yu and collaborators demonstrated the detection of cTnT by the enzyme-catalyzed 3,3′,5,5′-tetramethylbenzidine (TMB) reaction, which led to the formation of the detectable TMB^2+^ and enabled the chromogenic analysis of cTnT, and, moreover, a SERS quantitative analysis due to the final enzymatic product of Raman activity [35]. Figure 2a presents the absorption spectra of the TMB^2+^ with different cTnT concentrations spanning from 2 to 320 pg/mL. The formation of the final TMB^2+^ is correlated with the amount of the target analyte, thus by monitoring the absorbance of the enzymatic product, a LOD of 8 pg/mL can be established, which is in good agreement with the colorimetric observations (Figure 2a, inset). Moreover, the authors took one step further and combined the ELISA technique with SERS by adding TMB^2+^ to a AuNP colloidal solution. Under the 633 nm laser excitation, the main TMB^2+^ vibrational bands were observed at cTnT concentrations below 8 pg/mL, achieving the even lower LOD of 2 pg/mL (Figure 2b). This study demonstrates the potential of the SERS technique to improve the already employed detection kits such as ELISA and expand its applicability field as an analytical tool in clinical diagnosis.

The SERS enhancement results from the EM and CM effects, which are conditioned by the composition, morphology, and aggregation state of the nanoparticles used. Metallic nanoparticles are known to be efficient SERS enhancers, however, their combined use with magnetic beads, which are able to capture and separate the target analytes thus inducing nanoparticle aggregation/conglomeration, can improve their performance. In this scientific context, cardiac biomarker immunoassays have been developed to use metallic nanoparticles as SERS nanotags and magnetic beads as capture probes. Such a SERS nanoplatform was fabricated and studied for the selective and sensitive detection of the cTnI cardiac biomarker [36]. The proposed immunoassay blends the excellent properties of AuNPs with the intriguing structure and easy conjugation of graphene oxide (GO) to fabricate SERS nanotags, which incorporate a Raman reporter, herein malachite green isothiocyanate (MGITC), and were further conjugated with rabbit polyclonal cTnI antibodies. To form the sandwich structure of the proposed immunoassay, magnetic beads were functionalized with mouse monoclonal cTnI antibodies to assemble the capture probe. cTnI concentrations ranging from 0 to 1000 ng/mL were tested and by monitoring the MGITC SERS vibrational bands, the LOD of the developed immunoassay was estimated at 5 pg/mL, much lower compared to the cut-off value of 1.1 ng/mL needed for the diagnosis of AMI [37]. Similarly, an efficient nanoplatform, which incorporates both nanoparticles and magnetic beads for the detection of the cardiac biomarkers was developed by Hu and colleagues, who proposed a sandwich type structure that allows for the simultaneous SERS detection of both cTnI and heart-type fatty acid-binding protein (H-FABP) [38]. Briefly, SERS immunoprobes were developed by embedding two Raman reporters, specifically 4-mercaptobenzonitrile and XP013, in between the gold core and Ag shell followed by their conjugation with the biomarker equivalent antibodies. The immunological interaction between the SERS immunoprobes, capture probes (biotinylated antibodies), and antigens of interest leads to the assembly of the sandwich type structure, and furthermore, to the specific and efficient detection of the target antigens. The streptavidin-conjugated magnetic beads play an important role in the separation and enrichment of the target antigens. The proposed immunoassay demonstrated high specificity and sensitivity, being able to detect both H-FABP and cTnI in ultralow concentrations such as 0.6396 and 0.0044 ng/mL, respectively, hence promoting its implementation for an accurate diagnosis of AMI. Later on, based on the same principle, the group achieved a LOD of 9.8 pg/mL of cTnI, which is considerably lower than the approved cTnI threshold for the diagnosis of cardiac diseases, by using core-shell nanotags and a magnetic separation based SERS immunoassay by employing 4-mercaptobenzoic acid as an active Raman reporter, thus achieving a diagnostic tool with advantages such as specificity, ultrasensitivity, reduced costs of fabrication, ease-of-use, and no sample pre-treatment with promising reliability and stability [39].

**Figure 3:**
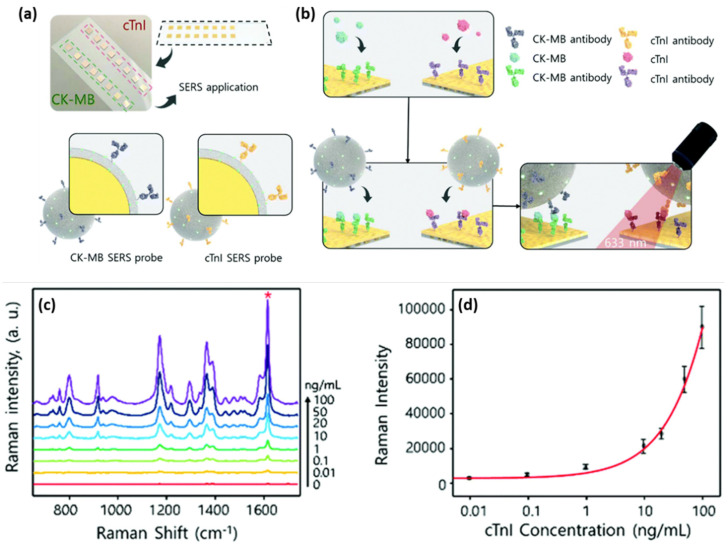
The schematic illustration of the (**a**) gold-patterned chip and SERS probes and (**b**) sandwich structure of the SERS immunoassay. (**c**) The SERS spectra recorded for increasing concentrations of cTnI ranging from 0 to 100 ng/mL and (**d**) the calibration curve of the 1615 cm^−1^ SERS band intensity as a function of the cTnI concentration on a logarithmic scale. Figure adapted from Cheng et al. [40].

The deposition of metallic nanoparticles onto solid substrates ensured the roughness of the metallic surface, thus enabling efficient SERS performances. The most common approach to fabricate efficient SERS substrates can be adapted and optimized in order to obtain more complex biosensing devices. Cheng et al. reported the fabrication of a SERS immunoassay with a sandwich type structure that efficiently combined a gold-patterned array chip with Au@Ag core-shell nanoparticle-based SERS probe for the specific and sensitive detection of dual cardiac biomarkers, specifically cTnI and creatine kinase-MB (CK-MB) [40]. The formation of the gold-patterned chip was realized by the deposition through immersion of AuNPs onto silicon wafers, which were then arranged on a glass slide and conjugated with cTnI and CK-MB monoclonal antibodies (Figure 3a,b). The SERS probes consisted of Au@Ag core-shell nanoparticles with the MGITC Raman reporter grafted on their surface, conjugated with polyclonal cTnI and CK-MB antibodies (Figure 3a). The capture of the target biomarkers was realized by the conjugated gold-patterned array chip followed by the addition of the SERS probes; thus, the sandwich type immunoassay was realized. Figure 3c shows the SERS spectra recorded for the cTnI concentrations ranging from 0 to 100 ng/mL, where the characteristic vibrational modes of MGITC are distinguishable, even for low antigen concentrations. To evaluate the LOD, a calibration curve was realized for the 1615 cm^−1^ MGITC characteristic vibrational band, hence the lowest detected cTnI concentration of 8.9 pg/mL was estimated (Figure 3d), which is 2- to 3-fold more sensitive compared to the currently employed techniques. Along with the proven high sensitivity of the developed biosensing, the authors also demonstrated its high specificity by performing selectivity tests against several proteins, thus promoting the reported assay as an efficient diagnostic tool of cardiac injuries.

The designs of immunosensor assays generally rely on a specific antigen–antibody recognition interaction, nonetheless, a new type of biomolecule, namely aptamers, has emerged and gathered a lot of attention lately due to their high susceptibility to specific biological molecules of interest, proving themselves to be promising for the development of sensitive, specific, and accurate immunosensors. Aptamers are synthetically isolated DNA or RNA-based single-stranded oligonucleotides, which enable the selective capture of target molecules due to their unique 3D folding ability. Regarding antibodies, they represent a more advantageous alternative in terms of production costs, chemical stability, and size. Furthermore, the efficient combination of aptamers with plasmonic nanomaterials, leading to the formation of the “aptasensors”, encourage and facilitate the interaction between the metallic surface and target analytes. SERS-based aptasensors have been demonstrated to effectively detect a wide range of chemical [41,42] and biological [43,44] target entities, proving the ability to substantially enhance the molecular recognition. For the sensitive and accurate detection of cardiac biomarkers and, more specifically, the cardiac troponin antigens (cTnI or cTnT), several aptasensor designs have been developed and proven to significantly decrease the LOD by exceeding the lower threshold values of the currently employed clinical testing kits such as ELISA. Lee and collaborators reported on an aptamer-immobilized gold nanoplate nanoplatform for the specific and accurate detection of cTnIs [45]. An atomically flat Au nanoplate was functionalized with an aptamer that has a strong affinity for the cTnI biomarker. The developed nanoplatform was tested both in the buffer solution and serum, reaching limits of detection of 100 aM (2.4 fg/mL) and 100 fM (2.4 pg/mL), respectively, thus proving a high sensitivity compared to the previously reported methods. Moreover, the authors further implemented the aptamer-immobilized Au nanoplate nanoplatform in the clinical diagnosis of AMI, being able to diagnose nine clinical samples showing better accuracy than ELISA. Furthermore, the combination of aptamers, Ag nanoparticles, and a magnetic element in the development of a label-free SERS biosensor led to a potential high-performance AMI diagnostic tool [46]. The applied strategy relies on the capabilities of an Fe_3_O_4_ magnetic core to induce magnetic aggregation, the sharp tips of flower-shaped Ag nanoparticles to generate SERS “hot-spots” and, implicitly, to enhance the SERS signal, and high affinity of an aptamer to specifically bind cTnI, leading to the direct detection of the cardiac biomarker. On the silica coated Fe_3_O_4_ core, the Ag nanoflowers were grown and further functionalized with the aptamer. Upon cTnI capture, SERS spectra were recorded using a 785 nm laser line. The vibrational “fingerprint” was identified and assigned specifically to the cTnI biomarker. The design of the developed biosensor allowed for the differentiation and precise assignment of the characteristic vibrational modes of the target analyte, even at the low concentration of 10^−11^ M (10 ng/mL), with the SERS spectrum having a comparable average intensity with the SERS spectrum of higher concentrated samples, indicating that the molecules tend to bind mainly to the tips of the nanostructures, hence, even though there is a lower concentration, a considerable amount of cTnI is placed in the hot-spot regions, enabling the detection of the biomarker at concentration ranges found in patients diagnosed with cardiac injury.

**Figure 4:**
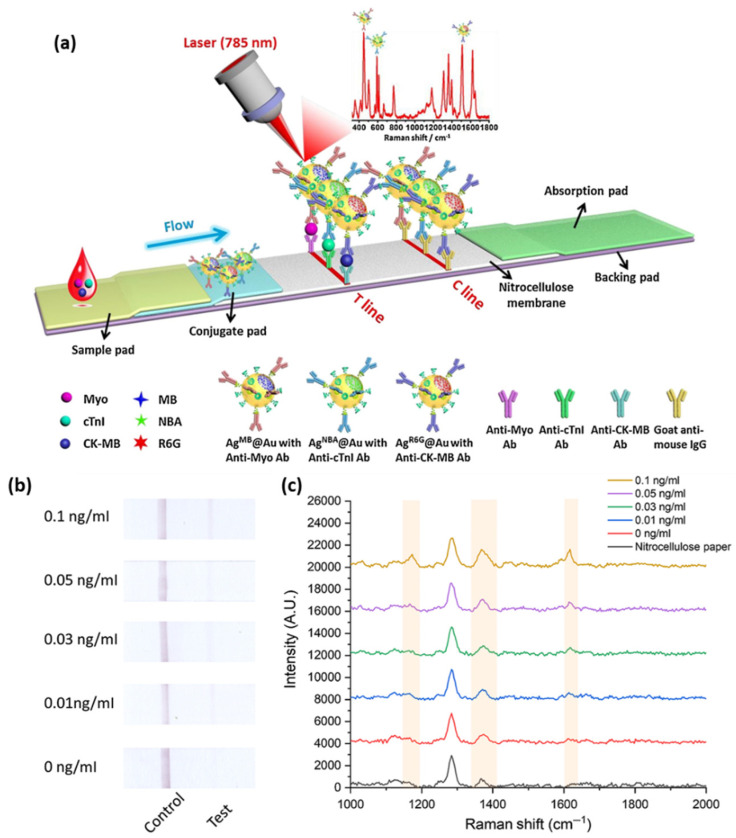
(**a**) The schematic illustration of the lateral flow assay for the detection of cardiac biomarkers on a single T-line. Figure adapted from Zhang et al. [51]. (**b**) Digital images of the paper-based fluidic platforms and (**c**) SERRS spectra from the test lines after the capture of different concentrations of cTnI via MGITC monitoring. Figure adapted from Tu et al. [52].

Lateral flow assays represent a technological progress in the context of POC devices due to their specificity, robustness, affordable costs, and multiplexing capabilities. Generally, the assay is based on the following configuration: (i) sample pad; (ii) conjugation pad; (iii) test (T) line; (iv) control (C) line; and (v) absorption pad, which ensures the lateral flow of the fluid. Lateral flow immunoassays have been developed and implemented in the detection of toxins and biomarkers including cardiac biomarkers [47,48]. Bai et al. focused on the evaluation of several bimetallic nanoparticles as SERS nanotags for the development of an efficient and sensitive later flow immunoassay for the detection of cTnI [49]. The SERS performance of: (i) Au; (ii) bimetallic Ag-Au; (iii) Au core-Ag shell; and (iv) rattle-like Au core-bimetallic Ag-Au shell nanoparticles, which were synthesized, and the Raman reporter Nile blue A (NBA) was grafted on their surface as well as embedded at the bimetallic interfaces. The latter SERS tag was proven both experimentally and theoretically to provide the best SERS enhancement. Furthermore, the SERS nanotags were conjugated with the monoclonal anti-cTnI detection antibodies and added to the separated conjugation pads, whereas, on the T-lines of the lateral flow assays, anti-cTnI capture antibodies were applied. Analyte-free human serum containing different cTnI concentrations was dropped on the sample pad. Optically, all four immunoassays performed approximately the same, even though the color intensity varied, with the determined LOD being a 5 ng/mL cTnI concentration for all assays. However, using the Au core-bimetallic Ag-Au shell nanoparticles, proven to exhibit a highly enhanced electromagnetic field localized in the gap in between the metallic surfaces, the SERS analysis allowed for the detection of a cTnI concentration as low as 0.09 ng/mL, which is 50 times lower than that shown by the visual assessment, thus has great potential for further optimization and implementation as a POC test in light of cardiac injury diagnosis. A lateral flow detection device was also envisioned by Zhang and colleagues, who specifically aimed to quantitatively and rapidly detect three cardiac biomarkers: cTnI, CK-MB, and myoglobin (Myo) [50]. The reported assay uses Ag@Au core-shell nanoparticles with a Raman active molecule, specifically NBA, encapsulated in between the two metallic surfaces to functionalize the conjugate pad. Prior to the coverage of the conjugate pad, the SERS nanotags were conjugated with the corresponding detection antibodies against the three cardiac target analytes. Furthermore, three separate T-lines were prepared by immobilizing the biomarkers to capture antibodies as well as goat anti-mouse IgG as the C-line. Mixtures of the target biomarkers at various concentrations were injected onto the sample pad, inducing the lateral flow of the probe and specific capture of the antigens as the sample passed the conjugate pad reaching the T-lines due to capillary forces. Through the Raman mapping of the T-lines using the SERS intensity of the band located at 592 cm^−1^ (characteristic vibrational band of the NBA-functionalized nanoparticles), LODs of 0.8, 0.7, and 1 ng/mL were determined for cTnI, CK-MB, and Myo, respectively. The developed lateral flow immunoassay was then validated for clinical applications by testing 50 serum samples collected from AMI diagnosed patients and comparing the obtained results with conclusions drawn from the employed FDA approved clinical chemiluminescence immunoassay. The two detection strategies showed good correlation, nonetheless, the developed SERS lateral flow assay demonstrated its superiority in terms of ease of use, low cost, and rapid analysis, thus promoting its further improvement and implementation in clinical applications. Later on, the authors progressed to the multiplexed detection of the three biomarkers on the same test line [51]. The reported assay schematically illustrated in Figure 4a uses the same Ag@Au core-shell nanoparticles, however, three different Raman active molecules (i.e., NBA, Methylene blue (MB) and Rhodamine 6 G (R6 G)) were embedded at the bimetallic interface to fabricate three SERS nanotags. The SERS nanotags as well as the T-line were conjugated with the corresponding detection antibodies. SERS spectra were recorded from the T line and the characteristic vibrational bands of the Raman reporters were monitored, proving the high specificity and sensitivity of the lateral flow assay by evaluating the LODs for cTnI, CK-MB, and Myo at 0.89, 0.93, and 4.2 pg/mL, respectively.

Similar to the above described biosensing device, Tu et al. reported the successful fabrication of a paper-based fluidic platform for the sensitive and specific detection of cTnI by integrating aptamers instead of antibodies as molecular recognition elements [52]. The T-line was conjugated with the specific cTnI primary aptamer whereas on the C-line, the reverse complementary single-stranded DNA of the cTnI secondary aptamer was immobilized. The nanoparticle system has a molecule, specifically, the Raman reporter malachite green isothiocyanate (MGITC), embedded, which is resonantly excited by the 638 nm laser line, hence, giving rise to the surface-enhanced resonance Raman scattering (SERRS). The SERRS nanotag is then conjugated with the cTnI secondary aptamer to form the final nanosystem. Taking advantage of the fact that SERRS is 10 to 100 times stronger in comparison with SERS, the developed paper-based biosensing device reached a LOD of 0.016 ng/mL (Figure 4b,c). Moreover, the assay provided improved stability to the temperature and pH variations as well as the opportunity to be implemented on site using a portable reader showing good sensitive capabilities.

The previously discussed studies employed spherical nanoparticles as SERS enhancers. Recently, a new type of SERS tag has emerged, namely the gap-enhanced Raman tags (GERTs), which are multi-layered structures that have been demonstrated to considerably enhance the overall SERS enhancement [53]. Khlebtsov and collaborators developed a lateral flow immunoassay for the detection of cTnI using as SERS the GERTs obtained by covering Au nanorod cores with Au shells and embedding the Raman reporter 1,4-nitrobenzenethiol (NBT) in the 1 nm gap that formed in between the core and shell [54]. To fabricate the immunoassay, the GERTs were conjugated with monoclonal anti-cTnI antibodies (IC4) and applied to the conjugation pad. For the T-line, anti-cTnI antibodies (IC19) were used and goat anti-mouse immunoglobulin (GAMI) antibodies were immobilized to form the C-line. The SERS performance of the proposed immunoassay was evaluated by the Raman mapping and recording of SERS spectra. First, the proposed GERTs were evaluated against other nanostructures such as nanorods, nanoshells, nanostars, and GERTs with spherical cores, and proven to be more efficient SERS tags by monitoring the NBT vibrational band assigned to symmetrical NO_2_ stretching (1300–1343 cm^−1^). Furthermore, the LOD of the GERT-based SERS lateral flow immunoassay was evaluated at 0.1 ng/mL, demonstrating its high sensitivity and accuracy to obtain reliable information regarding even minor cardiac events.

A rapid and sensitive microfluidic plasmonic paper-based device (μPAD) was reported by Lim et al. [55], who developed a nitrocellulose membrane card with separate test zones onto a paper substrate by employing a solid wax printer, thus allowing for a branched flow of the probe and, implicitly, simultaneous multiplex detection of three cardiac biomarkers (i.e., glycogen phosphorylase isoenzyme BB (GPBB), CK-MB, and cTnT). The strategy implies a sandwich type immunoassay, where the corresponding monoclonal antibodies are immobilized onto the printed nitrocellulose membrane to capture the target biomarkers, followed by polyclonal antibody conjugated nanoparticles such as Au and Ag nanospheres and Au nanourchins, which are used as optical labels to grant distinct colorimetric signals assigned to the different test zones that are then measured with reflectance detectors, phone cameras, and/or scanners. Through colorimetric detection, the fabricated μPAD reached the cut-off values of the clinically employed detection devices by reaching LODs of 0.5 ng/mL for CK-MB (cut-off 4–7 ng/mL) and 0.05 ng/mL for cTnT (cut-off 0.01–0.05 ng/mL), respectively. Furthermore, based on this work, the authors significantly improved the efficiency of their μPAD by employing the SERS technique [56]. The same sandwich type immunoassay procedure was used, however, prior to the conjugation with the corresponding polyclonal antibodies, onto the surface of the metallic nanostructures, where three different Raman reporter molecules were grafted. Hence, the LOD values of the SERS-based μPAD were evaluated to be 8, 10, and 1 pg/mL for GPBB, CK-MB, and cTnT, respectively, which were considerably lower than the cut-off values of the available testing kits.

The SERS technique is by itself advantageous due to its fingerprinting ability, which enables the specific detection and identification of the target biomarker while offering, at the same time, important information about the interaction between the capture probe and detected molecule. SERS-based biosensors are the object of PoC testing since they can be integrated with ease in different diagnostic devices, proving the miniaturization and portability of both the biosensor and detection instrumentation. Furthermore, since the vibrational fingerprint is unique to each molecule, SERS biosensors exhibit excellent multiplexing capabilities, allowing for the simultaneous detection of several target biomarkers, thus also answering the demands of precision medicine in AMI diagnosis. In the development of SERS biosensors, particular attention has been paid to avoid the masking of the SERS signal by the fluorescence of molecules, interfering analytes, or non-specific binding. Additionally, when testing complex biological fluids, a significant background signal can be generated, thus altering the test results.

## 4. Fluorescence-Based Immunosensors

**Figure 5:**
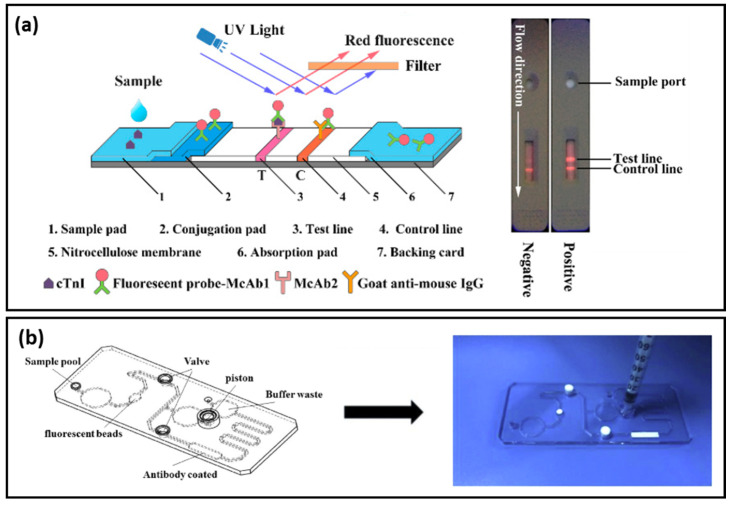
(**a**) The schematic illustration of the design of the LFIA (**left**) and digital images of negative and positive tests under UV-light (**right**). Figure adapted from Cai et al. [60]. (**b**) The schematic illustration and digital image of the design of the proposed microfluidic-based chip. Figure adapted from Huang et al. [64].

Fluorescence-based biosensors come as a valuable alternative to conventional colorimetric sensing tools, which, despite their accessibility in terms of cost, simplicity, and rapid visual read-out, present shortcomings related to their sensitivity and accuracy. On the other hand, fluorimetric analytical methods can achieve high sensitivity for real-time sensing applications and have been extensively exploited in the development of immunoassays [57]. As a matter-of-course, a great variety of fluorescence-based biosensors have been designed and optimized to efficiently and specifically detect cardiac troponin biomarkers for AMI diagnosis [58]. For instance, Song et al. designed a fluoro-microbead guiding chip (FMGC) that was able to operate as an effective sandwich immunoassay for cTnI [59]. The proposed FMGC can be divided into four immunoreaction regions, each having five imprinted Au patterns, which allow for multiple simultaneous assays to be carried out. The sandwich-like immunoassay relies on the conjugation of the FMGC surface with the specific anti-cTnI antibody using 3-3′-dithiobis-propionic acid N-hydroxysuccinimide ester, thus a self-assembled antigen-sensing monolayer was formed. After exposure to the cTnI containing sample, a detection antibody coupled to fluoro-microbeads was introduced and the cTnI concentration was assessed based on the number of biospecific fluoro-microbeads bound to the as-designed Au patterns of the FMGC. The biosensor was proven to be efficient in plasma samples containing 0.1 to 100 ng/mL cTnI, showing a LOD of 4.6 pM, which was 3-fold lower than the ELISA determinations in the same conditions. To improve the sensitivity even greater, the authors demonstrated that the use of the avidin–biotin interaction onto the detection fluoro-microbeads could lead to a LOD of 1.4 pM. Cai and collaborators efficiently combined immunochromatography with fluorescent microspheres to develop an accurate and reliable lateral flow immunoassay (LFIA), thus leading to an innovative and fast POC test that is able to detect cTnI [60]. LFIA designs are simple, easy-to-use, and fast testing methods, however, the analytical sensitivity and specificity still need to be addressed, especially in the case of cTnI, whose detection can be altered by the autofluorescence of interfering factors. To overcome these limitations, the authors proposed the synthesis of double layered fluorescent microspheres. Thus, approximatively 550 nm core-shell structures with Nile Red (NR) red fluorescent dye were used to differentiate between cTnI and the autofluorescence of plasma samples, embedded in the polymer. In Figure 5a, the left side shows the working principle of the proposed LFIA: the cTnI sample was dropped onto the sample pad and was driven due to the capillary forces to the conjugate pad containing the anti-cTnI labelled microspheres of McAb1, which specifically bind cTnI, followed by their capture on the anti-cTnI McAb2 functionalized nitrocellulose membrane depicted as the T-line. The fluorescent microsphere excess will attach to the C-line. The as-obtained test strip was placed under UV-light (Figure 5a-right side) and the fluorescence intensity was analyzed, showing a LOD of 16 pg/mL and limits of quantification of 87 pg/mL, which indicate the high sensitivity of the developed sandwich type immunoassay, offering a test result in less than 15 min. High-sensitivity cTnI tests require the detection of cTnI below 0.01 ng/mL, thus signal amplification is urgently needed in the development of such assays. A similar LFIA biosensor for cTnI was recently developed by Li and collaborators [61]. Quantum dot beads (QBs) coated in SiO_2_ (QBs@SiO_2_-COOH) exhibiting great stability and high fluorescence emission were generated in the presence of polyvinylpyrrolidone (PVP) to improve the sensitivity of LFIA. Compared to quantum dots (QDs), the QBs@SiO_2_-COOH showed a 1967-fold higher luminescence and better colloidal stability. Thus, QBs@SiO_2_-COOH was implemented as a capture element in the reported LFIA biosensor. Based on their fluorescence emission, a LOD of 36 pg/mL was obtained. Moreover, after the optimization processes, the proposed LFIA was demonstrated to simultaneously detect cTNI, CK-MB, and Myo; for the last two, the LOD were determined to be 0.25 and 0.54 ng/mL, respectively. The results were compared with the clinical results and showed good agreement, thus proving the versatility of the LFIA biosensor. Seo et al. proposed the labelling of the detection cTnI antibody with fluorescent molecules to develop a fast immunosensor [62]. The detection antibody was first biotinylated, followed by a polymerization process with fluorophore-conjugated streptavidin, and Dylight 650 and Alexa 647 were used as fluorescent molecules. Using scanning fluorometry, upon cTnI capture from the serum probes, the fluorescence emissions of the two dyes were studied. The LODs of 2 pg/mL for Dylight 650 and 7 pg/mL for Alexa 647 were established, thus high-performance fluorescent trackers were fabricated, revealing their true potential for AMI diagnosis compared to other conventional techniques. To improve the recorded fluorescence signal, Miao and collaborators reported on a nanozyme-linked immunosorbent assay, which relies on the use of graphitic carbon nitride QDs (g-C_3_N_4_ QDs) for the dual fluorescent-colorimetric detection of cTnI [63]. An organic substrate, as an efficient electron acceptor, was loaded with g-C_3_N_4_ QDs as exogenous fluorescent sources. The fluorescence emission could be quenched by the substrate, thus yielding a fluorescent line with two emission peaks under one wavelength illumination. The fluorescence changes were also visible to the naked eye. The detection of cTnI was presented as a proof-of-concept, demonstrating the capability of the nanozyme-linked immunosorbent assay to reach LODs of 0.413 pg/mL for radiometric fluorescence and 0.227 pg/mL for the visual read-outs, hence, proving the high sensitivity of the colorimetric detection along with the dual-modal detection abilities of the as-designed immunoassay.

Microfluidic devices have shown great potential in biomedical applications, benefiting from the advantage of the precise control of biochemical reactions inside the microfluidic channel, thus favoring accurate detection. A microfluidic device for the specific and accurate detection of high-sensitivity cTnI was reported by Huang et al. [64]. The proposed design could operate in the absence of the sample and conjugate pads while improving the precision of the chromatographic method by implementing Cy 5 fluorescent microspheres to which the cTnI antibodies were coupled. The as-developed microfluidic biosensor (Figure 5b) revealed a LOD of 5 pg/mL with a coefficient of variation of 2–4%. Furthermore, the fast response obtained within 10 min suggests its superiority to conventional chromatographic assays.

Another fluorescence-based assay is Förster resonance energy transfer (FRET) biosensing, which takes advantage of the antibody–antigen binding induced conformational changes and, implicitly, the generation of a measurable signal. In brief, FRET is a distance-dependent phenomenon occurring between two fluorescent molecules, the acceptor and donor, which are in close proximity with each other and, furthermore, exhibits a spectral overlap between the emission spectrum of the donor and absorption band of the acceptor. The transferred energy is non-radiative and resonant; hence the emission and adsorption of photons is not required. Nonetheless, the energy transfer can be quantified by determining the energy transfer efficiency. Given the fact that the Förster distance—intrinsic property of the fluorescent molecule pair—is in the range of many biological macromolecules, FRET has become a transduction method for biological interaction studies [65]. Grant et al. developed a FRET immunosensor for the detection of both cTnI and cTnT by measuring the morphological change in the antibody resulting from the antibody–antigen interaction as a shift in energy transfer [66]. The protein-based complexes induced a detectable change in fluorescence for cTnI and cTnT concentrations of up to 27 nM. Later, the same group optimized the design of the FRET biosensor for the detection of cardiac cTnI and placed it in a liquid core waveguide environment [67]. In their design, they employed QDs with an emission peak at 544 nm as donors and the Alexa 546 fluorophore as the acceptor. The as-obtained biosensor showed a highly sensitive and accurate performance, reaching an approximate cTnI LOD of 32 nM in phosphate buffered saline (PBS) and 55 nM in human plasma. Gogoi and Khan successfully integrated carbon dots (CDs) with molybdenum disulfide (MoS_2_) nanosheets for the efficient FRET-based sensitive detection of cTnT [68]. The MoS_2_ nanosheets bind to the anti-cTnT-conjugated with NIR-responsive CDs, hence the upconversion fluorescence is quenched. However, the specific capture of cTnT caused changes in the positions of the nano-couple, forcing the CDs to detach from the nanosheets and, implicitly, hindered the energy transfer from the CDs to MoS_2_. The proposed immunosensor was proven to reach a LOD of 0.12 ng/mL and a 0.38 ng/mL quantification limit, exhibiting a linear behavior for concentrations ranging from 0.1 to 50 ng/mL with a correlation coefficient of 0.99.

Recently, Tran and Ju reported on a fluorescence-based sandwich immunoassay that relied on the non-radiative coupling of fluorophores with surface plasmons, a phenomenon known as surface plasmon coupled emission (SPCE) [69]. The SPCE fluorescence chip was composed of bimetallic, Au, and Ag thin films that interacted with the antibody-conjugated Alexa 488 fluorophore. A 50-fold fluorescence enhancement was demonstrated for the as-developed SPCE chip; thus, the sensitivity of the assay was greatly improved, reaching a LOD of 21.2 ag/mL, the lowest reported to date.

Similar to SPR biosensors, fluorescence-based sensors have demonstrated their capability to efficiently detect cardiac biomarkers by labelling the capture molecule with fluorescent dyes or nanostructures. Their attractiveness lies in their simplicity and fast real-time response, which makes them excellent candidates for their integration with paper-based sensors and microfluidic chips, thus promoting their applicability in the PoC field. Nonetheless, a great amount of effort has been invested to further improve their sensitivity, which is related to the low quantum yields of fluorophores as well as their photostability.

## 5. Electrochemical Biosensors

Electrochemical (ETC) biosensors represent an important class of sensors due to their simplicity and low cost as well as several advantages such as specificity and sensitivity. Moreover, the ETC detection method offers portability and modularity, considering that the sensor can be easily miniaturized and integrated with another device, requires a minimal volume of sample, and has a short measurement time. Regarding the type of probed signal, the ETC sensors can be voltametric/amperometric, potentiometric, conductometric, or impedimetric. In recent years, electrochemistry has become a popular method to detect biomarkers while numerous efforts have been made to fabricate and implement novel ETC biosensors for the detection of cardiac troponins using different molecular recognition elements such as antibodies [70], specific peptides [71], and aptamers [72]. More importantly, the combination of ETC methods with different nanostructures (e.g., nanoparticles and nanocomposites) can increase the effective area of the working electrode and enhance the LOD, specificity, surface roughness, reactivity, immobilization efficiency, and electron transfer process.

**Figure 6:**
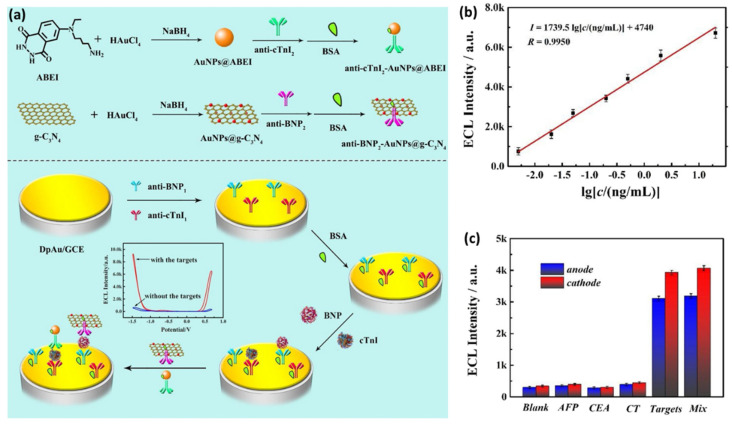
(**a**) The preparation of the secondary antibody composites and the fabrication of the immunosensor for dual detection. (**b**) Linearity curve between the ECL intensities and the logarithm of cTnI concentrations. (**c**) The ECL responses of the biosensor to interfering substances and the cTnI–BNP mix solution. Figure adapted from Zhao et al. [80].

ETC immunoassays are a particular type of biosensor that use antibodies as biological capture elements and quantitatively measure the electrical signal resulting from the binding event between the antibody and target molecule (i.e., the antigen). For example, an early report presented by Guo et al. in a novel quantitative ETC immunoassay for cTnI was based on the dual monoclonal “sandwich” detection principle by using a carbon paste electrode functionalized with the capture antibody IgG1 and the detection antibody IgG2, functionalized with the colloidal AuNP coated with Ag. Anodic stripping voltammetry detection assays revealed a linear range for cTnI concentrations from 1 to 20 ng/mL while the LOD achieved was 0.8 ng/mL [73]. Later, Zhou and collaborators developed an ETC immunoassay for the simultaneous detection of dual cardiac markers (i.e., cTnI and C-reactive protein) in a poly(dimethylsiloxane)–AuNP composite microfluidic chip. Their strategy involved the use of quantum dots labelled with antibodies and square-wave anodic stripping voltammetry detection in a microchannel via flow injection. For cTnI, the linear range of their assay was between 0.01 and 50 μg/L and the obtained LOD was approximately 5 amol in a 30 μL sample [74]. AuNPs were also electrodeposited by Saleh Ahammad et al. onto an indium tin oxide electrode and employed to detect cTnI via a “sandwich” of a highly-sensitive enzyme-based immune catalytic reaction by using a monoclonal antibody against cTnI and measuring the changes in the open circuit potential. A “sandwich” type molecular interaction occurred between the capture anti-troponin immobilized on the AuNP-modified electrode, cTnI, and horseradish-conjugated anti-troponin antibody as the detection protein. Using the proposed procedure, the authors reported a linear dependence between the potential changes and cTnI concentrations in the 1 to 100 ng/mL concentration range [75]. At the same time, a novel AuNP-based sandwich-type electrochemiluminescence (ECL) immunosensor was designed for the highly sensitive and selective detection of cTnI via electrochemical impedance spectroscopy (EIS) by using an electrode modified with streptavidin-coated AuNRs and N-(aminobutyl)-N-(ethylisoluminol)-functionalized AuNPs as labels for secondary antibodies [76]. The authors claimed that the electron-transfer process was facilitated by the streptavidin-coated AuNRs used as the immobilization matrix. The developed immunosensor exhibited a dynamic range from 2.5 to 10,000 pg/mL and allowed a LOD of 2 pg/mL. Later on, Bhalla et al. proved that colloidal citrate-capped AuNPs could successfully exhibit a dual role when deposited onto screen printed electrodes as a matrix for direct antibody immobilization and transduction properties [77]. Specifically, the authors proposed a one-step low-cost electrochemically controlled platform for the capacitance-based selective pH-dependent detection of cTnI in a label free manner with a LOD of 0.2 ng/mL. More recently, the label-free detection approach was also exploited by Liu et al., who presented an amperometric immunosensor based on AuNP–graphene oxide nanocomposites for the highly selective and sensitive detection of human cTnI. According to the authors, the employed nanocomposites helped improve the loading efficiency of the anti-cTnI capture antibody due to the large surface area while acting as a bridge for fast electron transfer, leading to increased sensitivity. The authors showed that the lowest concentration of cTnI detected in the buffer was 0.05 ng m/L, which was 100 times lower than that achieved by conventional methods, while the assay time was less than 5 min [78]. Meanwhile, Xiong and co-workers biosynthesized 3-mercaptopropionic acid-capped AgNPs and employed them to develop an ETC impedance cTnI immuno-transducer. Basically, the obtained AgNPs were covalently attached to an indium tin oxide–glass electrode functionalized with a self-assembled monolayer of 3-aminopropyltriethoxy silane. The immunoreaction of Ab-cTnI with complimentary cTnI conjugated to Ag-modified electrode led to the formation of kinetic impediment, which altered the charge transfer, forming the basis of the immunosensing [79]. Recently, Zhao et al. designed a novel potential-resolved ECL immunosensor based on AuNPs and two well-known ECL laminators (i.e., N-(aminobutyl)-N-(ethylisoluminol) (ABEI) and graphitic phase carbon nitride nanosheet (g-C_3_N_4_)) for the synchronously multiplexed immunoassay of two AMI biomarkers (i.e., brain natriuretic peptide (BNP) and cTnI) at the same interface, as schematically shown in Figure 6a [80]. First, the employed glassy carbon electrode with immobilized AuNPs was chemically modified to capture the anti-cTnI_1_ and anti-BNP_1_. Afterward, the antigens of cTnI and BNP and the secondary antibody composites were incubated step-by-step onto the electrode surface. Due to the formation of “sandwich” structures, both ABEI and g-C_3_N_4_ displayed an increase in the ECL signals, concomitant with increasing the concentration of cTnI and BNP, respectively, thus allowing for their simultaneous detection. The linearity curve between ECL intensities and cTnI concentrations, as shown in Figure 6b, revealed a LOD of 3.2 pg/mL for cTnI. Moreover, as shown in Figure 6c, the immunosensor showed excellent selectivity when possible interfering substances such as alpha fetoprotein (AFP), carcinoembryonic antigen (CEA), and calcitonin (CT) were used.

The photoelectrochemical (PEC) immunoassay has emerged lately as a promising bioanalytical method, its principle based on the immunoreaction taking place at the interface of the photoelectrode, which serves as signal transducer to convert the photoirradiation into an electrical signal for output. The split-type PEC immunoassay proved to be able to avoid several disadvantages of the common strategies such as the damage of the biomolecules and possible interferences. Liao et al. recently proposed a split-type PEC immunoassay for the ultrasensitive detection of cTnI, combining Ag_2_S/ZnO nanocomposites as a photoelectrode with the photogenerated hole-induced chemical redox cycling amplification (CRCA) strategy. In their proposed “sandwich” type detection, the authors employed AuNPs, which were functionalized with anti-cTnI molecules and alkaline phosphatase. The immunosensor demonstrated a LOD of 3.0 × 10^−15^ g/mL and it was used to analyze cTnI in the human serum samples, proving to have great potential in real sample analysis [81].

In recent years, peptides have received a great deal of attention as molecular recognition elements due to several advantages including their small molecular size, low cost, simple synthesis, simplicity of the labelling process, and increased chemical, thermal, and environmental stabilities [82]. For example, Wang and colleagues developed a label-free EIS peptide-based biosensor for the highly sensitive detection of cTnI based on glassy carbon electrodes modified with AuNPs employed as a platform for peptide deposition. A special peptide (CFYSHSFHENWPS), chosen as a recognition element, was deposited through self-assembly onto the AuNPs. It was found that such a AuNP–glassy carbon electrode assembly could significantly reduce the background signal in EIS, at the same time enhancing the target EIS response. The proposed biosensing approach proved to be highly sensitivity, selective, and reproducible for the detection of cTnI with a LOD of 3.4 pg/mL [83]. On the other hand, a particular type of highly sensitive voltametric peptisensor for the detection of cTnI from human serum samples, without the use of another biomolecule, was recently developed by Negahdary and Heli [84]. Specifically, a triangular icicle-like Au nanostructure was first electrodeposited on the Au electrode to obtain a high-surface area transducer, followed by the immobilization of the chosen 13 amino acid affinity peptide through the C-terminus cysteine moiety. Finally, the ferro/ferricyanide couple was applied as a redox marker to detect and quantify the cTnI binding with the peptide. Differential pulse voltammetry allowed for the quantification of cTnI in the 0.01–5 ng/mL concentration range with a LOD of 0.9 pg/mL.

**Figure 7:**
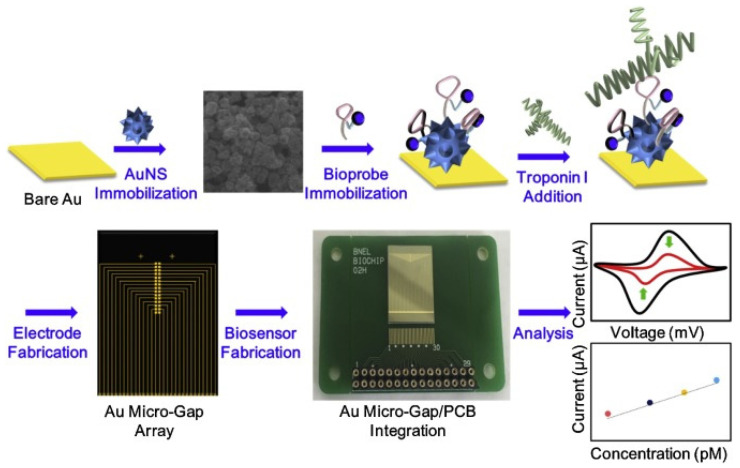
The schematic illustration of the fabricated ETC biosensor composed of a multifunctional DNA structure on the Au nanospike. Figure adapted from Lee et al. [89].

An aptasensor represents a compact analytical device employing an aptamer (single-stranded DNA or RNA molecules) as the biological recognition element, in association with a physiochemical transducer surface. Aside from their ability to carry genetic information, nucleic acids can also play a significant role in analytical monitoring, especially when combined with different nanostructured materials. For example, Negahdary et al. synthesized and employed Au nanodumbbells to fabricate a simple ETC aptasensor for the accurate low-cost detection of cTnI from blood serum samples [85]. The synthesized array of Au nanodumbbells over the Au electrode acted as a transducer for the immobilization of a 76-mer TnI aptamer used as the recognition element. With the obtained aptasensor, cTnI was detected in a linear range of 0.05–500 ng/mL with a LOD of 8.0 pg/mL. Moreover, the sensor showed a diagnostic sensitivity of 100% and a specificity of 85%. A different type of strategy was addressed for the first time by Jo et al., who fabricated a “sandwich” aptamer-based screen-printed carbon electrode modified with electrodeposited AuNPs for the highly sensitive amperometric detection of cTnI [86]. The amine-modified Tro4 aptamer was used as the capture probe while the hydrazine-modified Tro6 aptamer was employed as the detection probe. A LOD of 24 pg/mL was achieved in both the buffer and serum-added solution. The fabricated aptasensor exhibited excellent performance over the dynamic range of 0.024–2.4 ng/mL and a high sensitivity for cTnI over other proteins, having the potential to become an innovative tool for AMI diagnosis. A novel type of aptasensor for the ultrasensitive detection of cTnI was also recently reported by Qiao and collaborators [87]. The performance of the proposed sensing platform based on aptamer-molybdenum disulfide (MoS_2_) nanoconjugates was thoroughly evaluated by EIS in comparison to a second ETC aptasensor-based on core-shell Au@SiO2@Au nanoparticles. Significantly, the authors obtained a LOD of 1.23 pM for the aptamer-Au@SiO2@Au-based aptasensor and an even lower LOD of 0.95 pM for the aptamer-MoS_2_ nanosheet-based aptasensor, while both sensors were successfully tested for cTnI detection in human plasma samples. Later, the advantages of framework nucleic acids such as DNA nanotetrahedron (NTH) were exploited by Sun et al., who developed an enzyme-free ETC biosensor for cTnI detection, relying on the NTH-based dual-aptamer as the capture element and hybrid nanoelectrocatalysts containing Cu@Au NPs as a signal amplification probe. The proposed ETC aptasensor exhibited great analytical performance with a dynamic range of 0.05–100 ng/mL, a low LOD of 16 pg/mL, good repeatability, and high selectivity [88]. An interesting biosensor based on multi-functional DNA structure on Au nanospike (AuNS) was recently presented by Lee et al. [89]. Specifically, the authors developed an ETC label-free biosensor for cTnI using a multifunctional DNA 3 way-junction structure on a Au nanospike over a Au micro-gap electrode modified with a printed circuit board (PCB) chip, as shown in Figure 7, which was chosen to control each micro-gap panel selectively in order to detect low volumes of cTnI. The Au nanospikes were particularly employed in order to trigger the ETC signal enhancement.

Using cyclic voltammetry, the authors achieved a LOD of 1.0 pM in HEPES solution and 1.0 pM in 20% diluted human serum, respectively. More recently, 5 nm AuNPs were successfully employed as signal amplifiers to develop an ultrasensitive aptamer-based, sandwich-type surface plasmon enhanced ECL immunosensor for the detection of cTnI [90]. Using aptamer conjugated AuNPs as plasmon sources and the Tro4/Tro6 aptamers as the capture and detection probe, respectively, authors proved that the employed system showed ~5-fold enhancement compared to the system without AuNPs. Moreover, the proposed sensor displayed a LOD as low as 0.75 fg/mL.

The constant progress and improvement of electrochemical sensors has led to the development of ETC designs, which successfully integrate various technologies. Thus, fundamental features such as sensitivity and specificity have been improved. Along with the technological evolution, to some extent, the proposed biosensing chips exhibit versatility, miniaturization, portability, and reduced production costs. All of these advantages highlight the significant potential impact of ETC biosensors on health care and biomedicine once they are introduced on the market. On the way to their much-desired translation to the industry and, implicitly, the end-user, there are still some issues to be addressed. On one hand, the electrode interface’s resistance/stability to temperature and pH variations, especially after its surface modification with molecules, might limit the shelf life or storage conditions. The formation of by-products in the close vicinity of the electrode interferes with the target molecules, generating a misleading signal. On the other hand, the majority of the developed biosensors have been validated for the detection of one or two target biomarkers, thus the multiplexing detection capabilities of ETC biosensors remain a challenge.

## 6. Conclusions and Outlook

In this review, we summarized the significant developments realized throughout the progress of cardiac troponin biomarker detection tools, pointing out their strengths and limitations (Table 1). Despite the great variety of designs, methods, and technologies, individual or combined, that are currently employed in the diagnosis of AMI or proposed as alternatives, there remain a few challenges to overcome such as miniaturization, portability, fast analytical evaluation, reduced cost, and on-site biomarker detection. A great amount of continuous effort has been invested to improve the sensitivity, specificity, and accuracy of biosensors, however, the ultrasensitive, label-free, and fast cardiac troponin biomarker detection by an efficient high-throughput and affordable chip remains to be achieved. Moreover, the impressive results obtained for the detection of cardiac troponins support the further development and optimization of these biosensors for the detection of novel discovered diagnostic AMI biomarkers. The versatility of aptamers to selectively bind specific molecules could be exploited to realize diagnostic tools that are able to detect coagulation factors such as FXIII, which play a crucial role in the diagnosis and treatment of cardiac diseases [91,92]. The continuous effort invested in the design of diagnostic tools for AMI and cardiac diseases in general is impressive. Despite the fact that at the moment their translation to large-scale fabrication and the end-user has still not been reached, the advances made are paving the way for even more complex systems such as the much-desired implantable biosensors.

## Author Contributions

Conceptualization, M.F.; Writing—original draft preparation, A.C., I.M., A.-M.C., S.C., S.A. and M.F.; Writing—review and editing, M.F.; Supervision, M.F.; Project administration, M.F.; Funding acquisition, M.F. All authors have read and agreed to the published version of the manuscript.

## Figures and Tables

**Table 1:** Summary of all presented biosensors highlighting their strengths and limitations.

Detection Method	Target Biomarker	Sensor Type	Signal Transducers	LOD	Advantages	Limitations
**SPR/LSPR**	**cTnI**	Fiber-optic-based sensors	Au thin film	1.4 ng/mL [17]	Long-term stability, high specificity, fast, label-free PoC testing, anti-fouling capabilities	Sensitivity not sufficient for clinical diagnosis, multiplexing capabilities
		Au chips	Au thin film	68 pg/mL [18], 0.12 pg/mL [19]		
		Immunoassay in solution	HAuNPs	38 ng/mL [23]		
			AuNRs	10 ng/mL [25]		
			AuNRs aggregation	0.4 ng/mL [26]		
		Magnetic-field assisted immunoassay	HAuNPs-functionalized Au thin film and magnetic multi-welled carbon nanotubes	1.25 ng/mL [16]		
			AuNPs-functionalized Au thin film and core-shell Fe3O4@PDA nanohybrids	3.75 ng/mL [24]		
	**cTnT**	Au sensors	Molecularly imprinted polymer receptor	14.8 nM [20]		
			Self-assembled monolayer of conjugated polymers	100 ng/mL [21]		
			AuNPs	0.5 ng/mL [22]		
**SERS**	**cTnI**	Magnetic-field assisted immunoassay	Graphene oxide-conjugated AuNPs and magnetic beads	5 pg/mL [36]	Specificity, robustness, affordable costs, multiplexing capabilities, ease-of-use, rapid analysis, sensitivity	Fluorescence masked signal, background signal, non-specific binding
			Au@Ag core-shell nanoparticles and magnetic beads	4.4 pg/mL, 9.8 pg/mL [38,39]		
		Nanostructured sensing platform	Au-patterned array chip with Au@Ag core-shell nanoparticles	8.9 pg/mL [40]		
		Aptasensors	Au nanoplate	2.4 fg/mL [45]		
			Flower shaped Ag and Fe3O4 magnetic nanoparticles	10 ng/mL [46]		
		Lateral Flow Assay (LFA)	Au, Au-Ag, Au@Ag core-shell and rattle-like Au@Ag core-shell nanoparticles	90 pg/mL, 0.8 ng/mL, 0.89 pg/mL [49,50,51]		
			Aptamer-conjugated Au@Ag core-shell nanoparticles	16 pg/mL [52]		
			GERTs	0.1 ng/mL [54]		
	**cTnT**	Enzymatic Immunoassay	AuNPs colloidal solution	2 pg/mL [35]		
		Microfluidic paper-based device (µPAD)	AuNPs, AgNPs, and Au nanourchins	1 pg/mL [56]		
**Fluorescence**	**cTnI**	Fluoro-microbead guiding chip	Imprinted Au patterns and fluoro-microbeads	1.4 pM [59]	Fast, real-time sensing, high specificity, PoC testing	Sensitivity not sufficient for clinical diagnosis, multiplexing capabilities
		Lateral Flow Immunoassay (LFIA)	Double layered fluorescent microspheres	16 pg/mL [60]		
			Quantum dots beads	36 pg/mL [61]		
		Immunosensor in solution	Fluorophore-conjugated streptavidin	2 pg/mL, 7 pg/mL [62]		
		Nanozyme-linked immunosorbent assay	Quantum dots	0.413 pg/mL [63]		
		Microfluidic sensor	Fluorescent microspheres	5 pg/mL [64]		
		FRET biosensor	Protein-based complexes	27 nM [66]		
			Quantum dots	32 nM [67]		
		SPCE fluorescence chip	Bimetallic Au-Ag thin films	21.2 ag/mL [69]		
	**cTnT**	FRET biosensor	Protein-based complexes	27 nM [66]		
			Carbon dots	0.12 ng/mL [68]		
**Electrochemistry**	**cTnI**	ETC biosensor	carbon paste electrode and Ag-coated colloidal AuNPs	0.8 ng/mL [73]	Integrates various technologies, sensitivity, specificity, versatility, miniaturization, portability, reduced production costs	Electrode interface’s thermal and pH stability, damage of biomolecules, signal interferences, multiplexing capabilities
			Screen printed electrode modified with colloidal citrate-capped AuNPs	0.2 ng/mL [77]		
			Graphene oxide nanocomposites	0.05 ng/mL [78]		
			Indium tin oxide–glass electrode and biosynthesized AuNPs	1 ng/mL [79]		
		ETC microfluidic chip	Quantum dots and PDMS–AuNP composite	5 amol/30 µL [74]		
		Enzymatic Immunoassay	Indium tin oxide electrode functionalized with AuNPs	1 ng/mL [75]		
		ECL Immunosensor	Electrode modified with AuNRs and AuNPs	2 pg/mL [76]		
			AuNPs and ECL Laminators	3.2 pg/mL [80]		
		Photoelectrochemical immunoassay	Ag_2_S/ZnO nanocomposites as photoelectrodes	3 fg/mL [81]		
		Peptisensor	Glassy carbon electrode functionalized with AuNPs	3 pg/mL [83]		
			Triangular icicle-like Au nanostructure deposited on Au electrode	0.9 pg/mL [84]		
		Aptasensors	Au nanodumbbells and Au electrode	8 pg/mL [85]		
			Screen printed carbon Electrode modified with AuNPs	24 pg/mL [86]		
			Mos2 nanoconjugates; core-shell Au@sio2@Au nanoparticles	0.95 pM [87]		
			Hybrid Cu@AuNPs Nanoelectrocatalysts	16 pg/mL [88]		
			Au nanospike over Au micro-gap electrode	1 pM [89]		
			5 nm AuNPs	0.75 fg/mL [90]		

## Data Availability

Not applicable.
